# MAPK8IP2 is a potential prognostic biomarker and promote tumor progression in prostate cancer

**DOI:** 10.1186/s12885-022-10259-2

**Published:** 2022-11-11

**Authors:** Zhenhao Zeng, Wenrui He, Yi Jiang, Hao Jiang, Xiaofeng Cheng, Wen Deng, Xiaochen Zhou, Cheng Zhang, Gongxian Wang

**Affiliations:** 1 Department of Urology, The First Affiliated Hospital of Nanchang University, Nanchang, 330000 China; 2 Jiangxi Institute of Urology, Nanchang, 330000 China; 3 Department of Urology, The Second Affiliated Hospital of Nanchang University, Nanchang, 330000 China; 4 Department of Ultrasound Medicine, The Second Affiliated Hospital of Nanchang University, Nanchang, 330000 China

**Keywords:** MAPK8IP2, Prostate cancer, Methylation, Prognosis, Tumor progression

## Abstract

**Background:**

MAPK8IP2 is one of the JNK-interacting proteins (JIPs) family members, and is involved in the regulation of the JNK and P38 MAPK signaling pathways. MAPK8IP2 has been reported to be closely associated with several cancers. However, the biological function of MAPK8IP2 in prostate cancer (PCa) remains unclear.

**Methods:**

MAPK8IP2 expression in PCa and subgroups of PCa was analyzed by public databases. The prognostic role of MAPK8IP2 in prostate cancer was analyzed using the Cox regression method. The potential mechanism by which MAPK8IP2 affects PCa progression was investigated by utilizing public data, including genetic alteration, DNA methylation, m6A methylation, and immune infiltration data. We further performed in vitro assays to validate the effect of MAPK8IP2 on PCa cell proliferation, migration and invasion.

**Results:**

MAPK8IP2 is highly expressed in PCa tissues. Overexpression of MAPK8IP2 is associated with adverse clinicopathological factors and a poor prognosis in PCa. Receiver operating curve analysis showed that MAPK8IP2 can distinguish PCa tissues from non-PCa tissues with a certain accuracy (AUC = 0.814). The MAPK8IP2 genetic alteration rate was 2.6% and MAPK8IP2 alterations correlated with a poor prognosis. We also found that CDK12 and TP53 mutations were associated with MAPK8IP2 expression. The DNA methylation level of MAPK8IP2 was higher in primary tumors than in normal tissues, and the high MAPK8IP2 DNA methylation group of PCa patients had poor survival. Enrichment analysis indicated that MAPK8IP2 was involved in the MAPK signaling pathway. In vitro, knockdown of MAPK8IP2 inhibited PCa cell proliferation, migration and invasion.

**Conclusion:**

MAPK8IP2 is a potential target for PCa treatment and can serve as a novel biomarker for PCa diagnosis and prognosis evaluation.

**Supplementary Information:**

The online version contains supplementary material available at 10.1186/s12885-022-10259-2.

## Introduction

Prostate cancer (PCa) is the second most common malignant disease and the fifth leading cause of death in male cancer patients, accounting for nearly 3.8% of deaths and 7.3% of new cancer cases worldwide in 2020 [1]. PCa can be effectively treated by surgery, radiotherapy and hormone therapy [2]. However, tumor metastasis or castration resistance leads to a poor prognosis. Although the prognosis of prostate cancer patients has improved with the development of prostate cancer diagnosis and treatment strategies, it remains unsatisfactory [3]. Thus, there is a need for further research to explore new biomarkers to predict the occurrence and progression of prostate cancer.

The mitogen-activated protein kinase (MAPK) signaling pathway is one of the most widely studied signal transduction pathways. As one of the core members of the MAPK family, c-Jun NH2-terminal kinase (JNK) is implicated in cell migration, differentiation, proliferation, and apoptosis, and is also closely related to tumor generation and progression [4, 5]. JNK-interacting protein (JIP) plays a critical role in the MAPK signaling pathway [5].

Mitogen-activated protein kinase 8 interacting protein 2 (MAPK8IP2) (also known as JIP2) is located on chromosome 22 (22q13), and is involved in the regulation of the JNK and P38 MAPK signaling pathways [5]. MAPK8IP2 has a similar structure as JIP1, including an SH3 domain, a PTB domain, and a JNK-binding domain [6, 7]. Northern blot analysis demonstrated that MAPK8IP2 is expressed not only in the brain but also in other human tissues, such as the pancreas, ovary, and prostate [6]. Although studies have shown that the JNK and P38 MAPK pathways are associated with tumors [8], only a few studies have reported on the role of MAPK8IP2 in cancer. Recently, it was founded that MAPK8IP2 may be related to the prognosis of the glioblastoma and pancreatic cancer [9, 10]. Furthermore, MAPK8IP2 was identified to be associated with the progression of cervical cancer [11]. However, the function of MAPK8IP2 in PCa is unclear.

In this study, we analyzed the expression of MAPK8IP2 in PCa samples from The Cancer Genome Atlas (TCGA) and Gene Expression Omnibus (GEO) databases. Then, we evaluated the association between the expression level of MAPK8IP2 and clinicopathological parameters in PCa and assessed the diagnostic and prognostic value of MAPK8IP2 for PCa. In addition, to determine the potential molecular mechanism of MAPK8IP2 in prostate cancer, we used various databases to analyze the relationship of MAPK8IP2 expression with immune infiltration, gene mutation, m6A modification, and DNA methylation and explored the gene and functional network correlated with MAPK8IP2 expression. Finally, we confirmed that knockdown of MAPK8IP2 inhibited the proliferation, migration and invasion of PCa cell lines in vitro. Our study uncovered the important role of MAPK8IP2 in PCa and offers a novel potential biomarker for improving the diagnosis and prognosis of PCa.

## Materials and methods

### Expression and clinical correlation analysis of MAPK8IP2

Data for the TCGA and Genotype-Tissue Expression (GTEx) cohorts of the UCSC Xena database(https://xena.ucsc.edu/) [12] were used to analyze the expression of MAPK8IP2 in 33 types of human cancer.

Then, the expression of MAPK8IP2 and the relationship between MAPK8IP2 expression level and clinicopathological factors of prostate cancer patients were analyzed by using PRAD data from TCGA database (https://portal.gdc.cancer.gov/). The GSE70768 dataset from the GEO database (https://www.ncbi.nlm.nih.gov/gds) was also used to analyze the expression of MAPK8IP2 in PCa. In addition, we compared the expression of MAPK8IP2 in tumor tissues with normal tissues using the Human Protein Atlas (HPA) database(https://www.proteinatlas.org/) [13, 14].

### Genetic alteration and methylation analysis of MAPK8IP2

The genetic alterations of MAPK8IP2 in PCa were assessed using three datasets (MCTP, Nature 2012; SU2C/PCF Dream Team, PNAS 2019; TCGA, PanCancer Atlas) containing survival data in cBioPortal (www.cbioportal.org) [15, 16]. We used the muTarget (https://www.mutarget.com/) [17] platform to identify gene mutations altering MAPK8IP2 expression level and to identify the expression changes of genes associated with MAPK8IP2 mutation.

We analyzed the correlation between MAPK8IP2 and DNA methylation using the TCGA(PRAD) dataset from the UCSC Xena database. The MAPK8IP2 promoter methylation profile based on sample types, patient age, patient race, and nodal metastasis status was assessed using the TCGA(PRAD) dataset in the UALCAN database (http://ualcan.path.uab.edu/) [18]. Then, the value of MAPK8IP2 DNA methylation level as a survival prediction and diagnosis tool for prostate cancer was assessed based on the DNMIVD database (http://www.unimd.org/dnmivd/) [19].

Additionally, the relationship between MAPK8IP2 and m6A RNA methylation -related genes was also analyzed in PRAD samples from the TCGA database, and the differential expression of m6A RNA methylation-related genes between the low and high MAPK8IP2 expression groups was assessed. Twenty-one m6A RNA methylation-related genes [20] were included in the study.

### Immune infiltration analysis of MAPK8IP2

The single sample gene set enrichment algorithm (ssGSEA) from the R package “GSVA” [21] was used for immune infiltration analysis for PCa in the TCGA (PRAD) dataset. In addition, the association of MAPK8IP2 expression with the infiltration levels of twenty-four immune cells was evaluated. These 24 immune cell types are based on the literature reports [22].

### Protein–protein interaction (PPI) analysis and enrichment analysis of MAPK8IP2-related genes

We employed the STRING database (https://cn.string-db.org/) [23] to construct the PPI network. An interaction score > 0.9 was set to obtain the protein that interacts with MAPK8IP2. Nine genes interacting with MAPK8IP2 were obtained for Gene Ontology (GO) analysis and Kyoto Encyclopedia of Genes and Genomes (KEGG) analysis [24, 25]. The R package “ClusterProfiler” was used to GO and KEGG analysis [26]. Then, we used the TCGA (PRAD) dataset to analyze the correlation between MAPK8IP2 and nine partner genes and performed Kaplan–Meier analysis for patients grouped according to the expression of the nine partner genes. The differentially expressed genes correlated with MAPK8IP2 and MAPK8IP3 were analyzed via the LinkedOmics database (http://www.linkedomics.org/login.php) [27].

### Cell culture and transfection

The human normal prostate epithelial cell lines (RWPE-1 and HPrEC) and the human PCa cell lines (PC3, DU145, 22RV1, LNCaP, and VCaP) were obtained from ATCC. PC3 cells were grown with F12K medium (Gibco, USA). HPrECs were maintained in prostate epithelial cell basal medium (ATCC, USA). RWPE-1 and VCaP cells were cultured in DMEM (Gibco, USA). DU145, 22RV1, and LNCaP cells were maintained in RPMI-1640 medium (Gibco, USA). All the cells were grown in media with 10% FBS (Gibco, Australia) at 37 °C in a 5% CO2 incubator. MAPK8IP2 siRNA was synthesized by RiboBio (Guangzhou, China). The siRNA sequence was as follows: siMAPK8IP2: 5′-GCCATTTCTTCCAGATGAA-3′. Transfection was performed with Lipofectamine 2000 (Invitrogen, Thermo Fisher Scientific) according to the manufacturer’s instructions.

### Quantitative real-time PCR (qRT–PCR)

Total RNA was extracted by TRIzol reagent (CWBIO, China) and reverse transcribed into cDNA using the First-Strand cDNA Synthesis kit (Transgen, AT341-01) according to the manufacturer’s protocols. TransStart Green qPCR SuperMix (Transgen, AQ101-01) was used for qRT–PCR. Based on the 2^-∆∆CT^ method and using β-actin as a reference gene, relative expression was calculated. The primer sequences were as follows: β-actin: forward, 5′-TCTCCCAAGTCCACACAGG-3′ and reverse, 5′-GGCACGAAGGCTCATCA-3′; MAPK8IP2: forward, 5′-CGCTGCAGCCATTT.

CTTCC-3′ and reverse, 5′-ACTCCTGGGAGACAAAGACG-3′.

### Cell proliferation assays

The proliferation of cells was tested with 5-ethynyl-2′-deoxyuridine (EdU) and Cell Counting Kit 8 (CCK-8). Two days after siRNA transfection, PCa cells were seeded in 96-well plates for EdU and CCK-8 assays. PC3 (1 × 10^4^ cells/well), DU145 (1× 10^4^ cells/well), and 22RV1 (3 × 10^4^ cells/well) cells were seeded in 96-well plates, After the cells were cultured for 1 day, EdU staining was performed following the EdU kit (RiboBio, China) manufacturer’s guidelines. Cell proliferation was observed using fluorescence microscopy, and the percentage of EdU-positive cells was calculated. PC3 (8 × 10^3^ cells/well), DU145 (6× 10^3^ cells/well), and 22RV1 (5 × 10^4^ cells/well) cells were seeded in 96-well plates, 100 μl of medium containing 10% CCK-8 (APExBIO, USA) was added to each well at 24 h, 48 h, 72 h, and 96 h. After incubation for 2 h at 37 °C, the OD values at 450 nm were measured by a microplate reader.

### Transwell assay

Transwell chambers (8 μm pores, Corning, USA) were used for migration and invasion experiments. Then, 600 μl of complete medium containing 20% FBS was added to the bottom chamber. The transfected PC3 (6 × 10^4^ cells/well), DU145 (5 × 10^4^ cells/well), and 22RV1 (1.2 × 10^5^ cells/well) cells were seeded in the upper chamber in 200 μl FBS-free medium for cell migration (without Matrigel) and invasion (with Matrigel). After being cultured for 48 h (for PC3 and 22RV1 cells) or 24 h (for DU145 cells), cells that passed through the transwell membrane were fixed, stained and observed under a microscope.

### Western blot assay

Total protein was extracted with RIPA lysis buffer (APExBIO, USA) containing protease and phosphatase inhibitors and quantified with a BCA kit (CWBIO, China). Then, 10% SDS-PAGE was used to separate the proteins, and the transfer of proteins to PVDF membranes was performed. The membranes were incubated overnight at 4 °C with primary antibodies. The primary antibodies included GAPDH (Servicebio, China, 1:1000) and MAPK8IP2 (Santa Cruz, USA, 1:200).

### Statistical analysis

Statistical analysis of data from the TCGA and GEO databases was performed using R (v 3.6.3). MAPK8IP2 functional experimental data were statistically analyzed using GraphPad Prism (v 8.0.1). Continuous variables are summarized as the mean (SD), and categorical variables are shown as the number (%). The chi-squared test, Fisher’s test, and Mann-Whitney U test were used to analyze the association between MAPK8IP2 expression levels and clinicopathological factors. To identify significant differences between groups of MAPK8IP2 functional experiments, Student’s t-test, and ANOVA were used. Spearman’s rank test was used to assess the correlation between the expression of two genes. Univariate and multivariate Cox regression analyses revealed significant predictors of poor overall survival (OS) and short progression-free interval (PFI). Estimating OS and PFI were performed using Kaplan–Meier analysis, and the statistical significance was evaluated by log-rank test. The predictive ability of MAPK8IP2 to predict PCa outcomes was assessed by receiver operating characteristic (ROC) curve analysis. *p* <  0.05 was considered to indicate statistical significance.

## Results

### The expression level of MAPK8IP2 in PCa

**Fig. 1:**
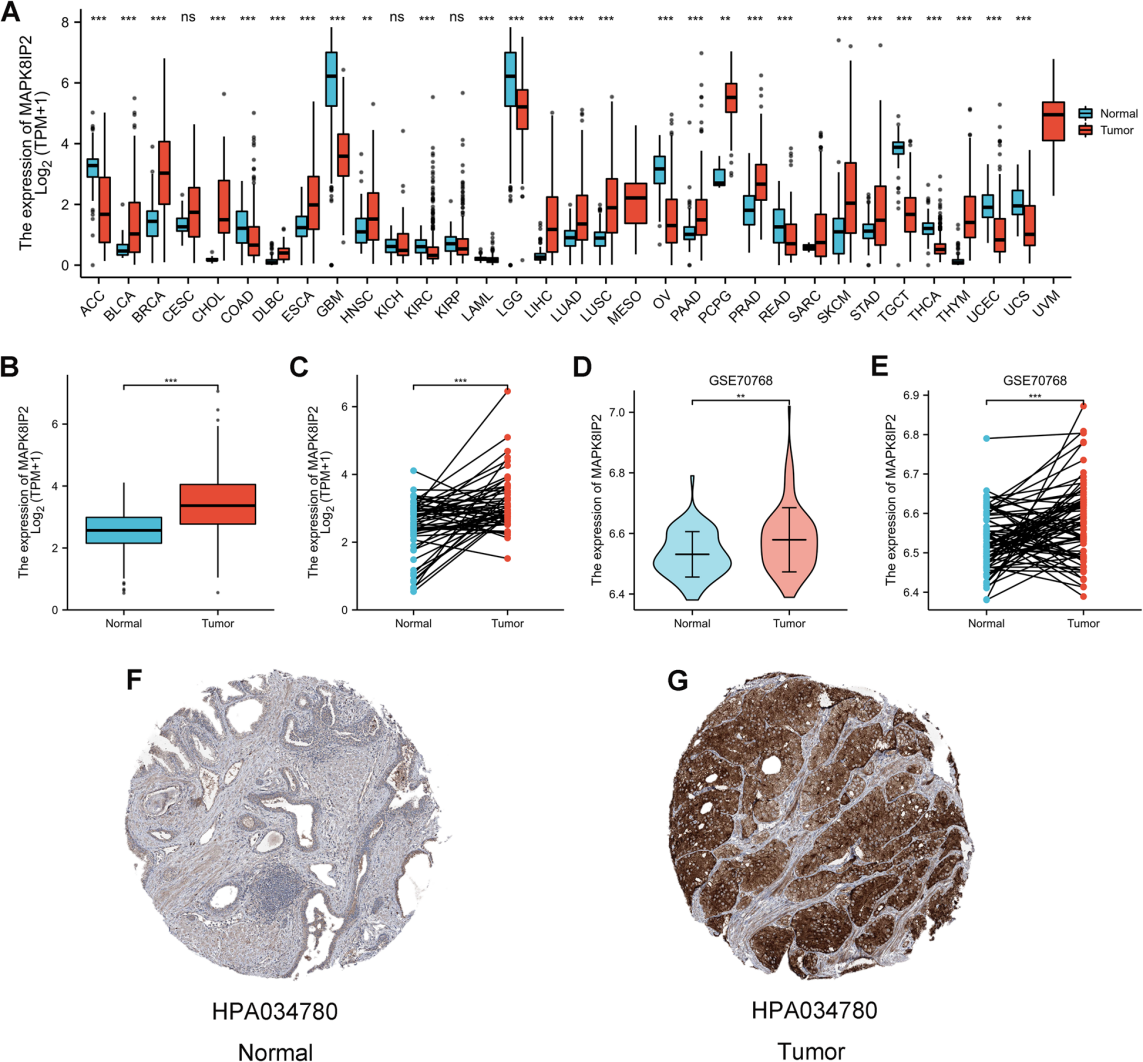
The expression of MAPK8IP2 in prostate cancer and pan-carcinoma. **A** MAPK8IP2 expression in unpaired tumor tissues and normal tissues of 33 types (TCGA and GTEx). **B**-**E** MAPK8IP2 expression levels in prostate cancer tissues and normal prostate tissues of TCGA (**C**) and GSE70768 (**D**, **E**) (paired and unpaired samples). **F**, **G** Immunohistochemical staining was used to evaluate the protein expression of MAPK8IP2 in normal tissue (**F**) and tumor tissue (G). *p < 0.05, **p < 0.01, ***p < 0.001

We used TCGA (tumor and normal data) and GTEx (normal data) cohorts to analyze the expression of MAPK8IP2 in 33 cancer types (tumor vs. normal) (Fig. 1A). The results showed significantly higher expression of MAPK8IP2 in 15 types of human cancer, including prostate adenocarcinoma (PRAD), breast invasive carcinoma (BRCA), and bladder urothelial carcinoma (BLCA) (cancer tissues vs. normal tissues). Then, we further evaluated MAPK8IP2 expression in prostate cancer by analyzing TCGA (PRAD) and GEO (GSE70768) datasets, [the TCGA included unpaired samples (52 normal tissues vs. 499 tumor tissues) (Fig. 1B) and paired samples (52 pairs) (Fig. 1C); GSE70786 included unpaired samples (73 normal tissues vs. 125 tumor tissues) (Fig. 1D) and paired samples (73 pairs) (Fig. 1E)]. In the analysis of paired and unpaired samples, MAPK8IP2 expression was significantly lower tin normal tissues than in tumor tissues. Moreover, MAPK8IP2 protein expression in tumor and normal tissues was analyzed via the HPA database. We found positive staining for MAPK8IP2 in most PCa tissues. As shown Fig. 1F (Patient ID: 2932) and Fig. 1G (Patient ID: 525), MAPK8IP2 staining was significantly higher tin PRAD tissue than in normal tissue.

### High expression of MAPK8IP2 was associated with adverse clinicopathological factors and a poor prognosis in PCa

**Fig. 2:**
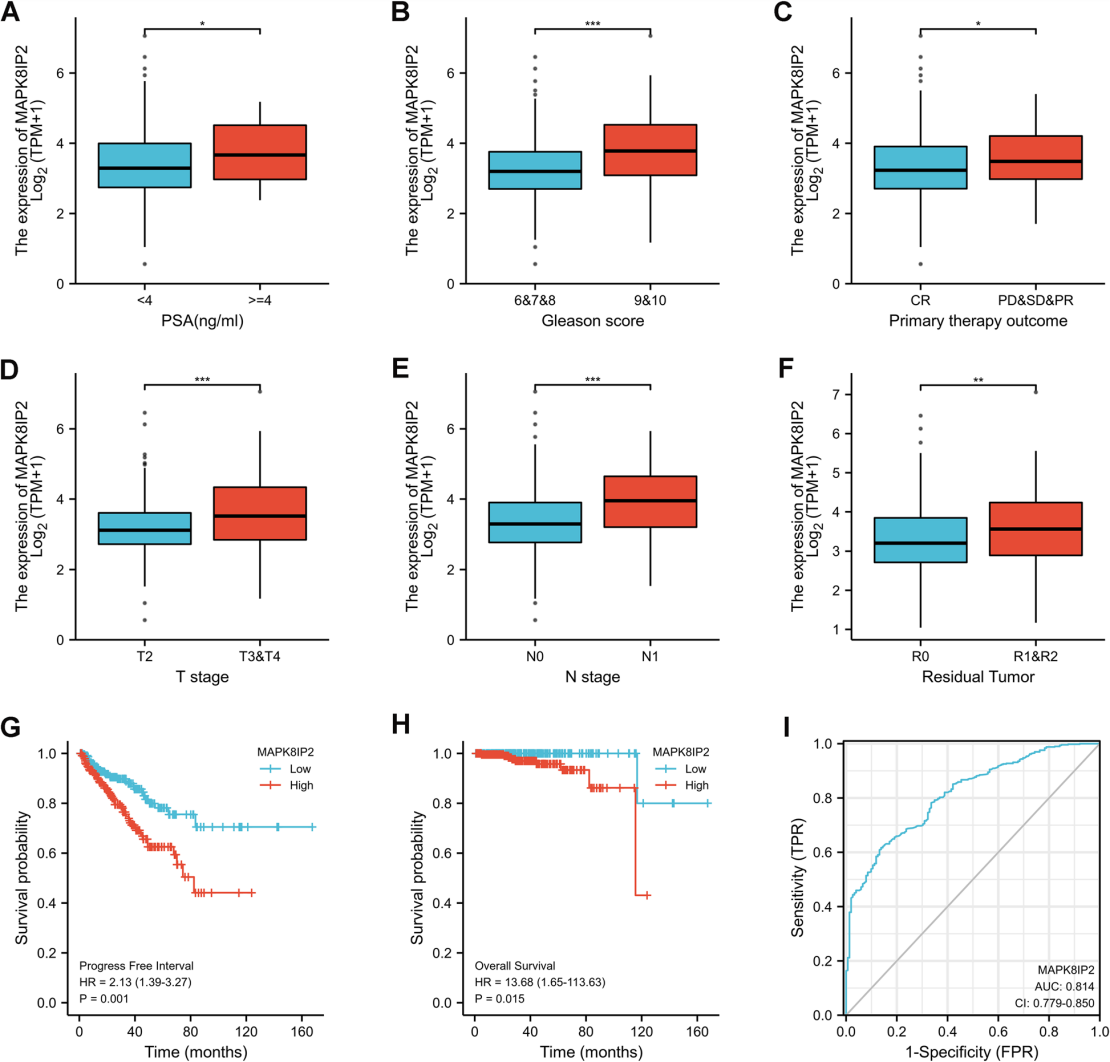
The association of MAPK8IP2 expression with clinicopathological factors in PCa (TCGA database). **A**-**F** MAPK8IP2 expression levels were significantly associated with PSA (**A**), Gleason score (**B**), primary therapy outcome (**C**), T stage (**D**), N stage (**E**), and residual tumor (**F**). **G**, **H** Kaplan–Meier analysis was performed to estimate the PFI (**G**) and OS (**H**) between the low and high MAPK8IP2 expression groups. **I** ROC curves were used to assess the predictive ability of MAPK8IP2 for PCa. CR: complete response; PD: progressive disease; SD: stable disease; PR: partial response; PFI: progression-free interval; OS: overall survival; ROC: receiver operating characteristic; *p < 0.05, **p < 0.01, ***p < 0.001

According to the expression levels of MAPK8IP2, we categorized the 499 PCa patients in the TCGA (PRAD) dataset into low (*n* = 249) and high (*n* = 250) expression groups according to the median value. The expression of MAPK8IP2 was significantly correlated with Gleason score (*P* <  0.001), T stage (*P* <  0.001), N stage (*P* = 0.002), primary therapy outcome (*P* = 0.048), and residual tumor (*P* = 0.009), but not with age, prostate specific antigen (PSA), M stage, race, and zone of origin, as shown in Table 1. Furthermore, patients were grouped according to clinicopathological factors, and we found that patients with PSA (≥ 4 ng/ml), Gleason score (9-10), primary treatment outcome (progressive disease (PD)/stable disease (SD)/partial response (PR)), T stage (T3-T4), N stage (N1) and residual tumor (R1-R2) showed higher levels of MAPK8IP2 expression than patients with other features, as shown in Fig. 2A-F.

**Table 1:** The correlation between MAPK8IP2 expression level and clinicopathological factors in PCa

Factors		Low expression of MAPK8IP2	High expression of MAPK8IP2	*P* value
**n**		**249**	**250**	
**Age, n (%)**	**≤60**	**118 (23.6%)**	**106 (21.2%)**	**0.303**
	**> 60**	**131 (26.3%)**	**144 (28.9%)**	
**PSA(ng/ml), n (%)**	**< 4**	**217 (49.1%)**	**198 (44.8%)**	**0.181**
	**≥4**	**10 (2.3%)**	**17 (3.8%)**	
**Gleason score, n (%)**	**6-8**	**198 (39.7%)**	**159 (31.8%)**	**< 0.001**
	**9-10**	**51 (10.2%)**	**91 (18.2%)**	
**T stage, n (%)**	**T2**	**116 (23.6%)**	**73 (14.8%)**	**< 0.001**
	**T3-T4**	**128 (26.0%)**	**175 (35.5%)**	
**N stage, n (%)**	**N0**	**181 (42.5%)**	**166 (39%)**	**0.002**
	**N1**	**25 (5.9%)**	**54 (12.7%)**	
**M stage, n (%)**	**M0**	**229 (50%)**	**226 (49.3%)**	**0.623**
	**M1**	**1 (0.2%)**	**2 (0.4%)**	
**Primary therapy outcome, n (%)**	**CR**	**183 (41.8%)**	**158 (36.1%)**	**0.048**
	**PD/SD/PR**	**41 (9.4%)**	**56 (12.8%)**	
**Race, n (%)**	**Asian**	**4 (0.8%)**	**8 (1.7%)**	**0.522**
	**Black or African American**	**29 (6%)**	**28 (5.8%)**	
	**White**	**206 (42.6%)**	**209 (43.2%)**	
**Residual tumor, n (%)**	**R0**	**175 (37.4%)**	**140 (29.9%)**	**0.009**
	**R1**	**61 (13%)**	**87 (18.6%)**	
	**R2**	**3 (0.6%)**	**2 (0.4%)**	
**Zone of origin, n (%)**	**Central Zone**	**3 (1.1%)**	**1 (0.4%)**	**0.174**
	**Overlapping / Multiple Zones**	**46 (16.7%)**	**80 (29.1%)**	
	**Peripheral Zone**	**64 (23.3%)**	**73 (26.5%)**	
	**Transition Zone**	**4 (1.5%)**	**4 (1.5%)**	

*PD* Progressive disease, *SD* Stable disease, *PR* Partial response, *CR* Complete response

We further performed Kaplan–Meier analysis to estimate the relationship between MAPK8IP2 expression and survival. The results indicated that high expression of MAPK8IP2 was significantly associated with short PFI (Fig. 2G) and OS (Fig. 2H) in PCa patients.

In addition, Cox regression analysis was used to determine whether MAPK8IP2 was an independent prognostic factor for predicting PFI and OS. Univariate Cox analysis associated with PFI showed that T stage, N stage, Gleason score, PSA, primary therapy outcome, residual tumor, and MAPK8IP2 affected PFI (Table 2). Multivariate Cox analysis demonstrated that Gleason score 9-10 (HR, 2.375; 95% CI 1.382-4.082; *P* = 0.002), primary treatment outcome PD/SD/PR (HR, 3.586; 95% CI 2.026-6.348; *P* <  0.001), and high MAPK8IP2 expression group (HR, 1.690; 95% CI 1.045-2.733; *P* = 0.032) were significant risk factors for PFI (Table 2). As shown in Table 3, the univariate Cox analysis showed that M stage, Gleason score, PSA, primary therapy outcome, and MAPK8IP2 were associated with OS.Multivariate Cox analysis also showed that high MAPK8IP2 expression group (HR, 1.690; 95% CI 1.045-2.733; *P* = 0.032) was a significant independent risk factors for OS, besides M1 stage.

**Table 2:** Univariate and multivariate Cox regression analyses of the factors predict progression-free interval in PCa

Factors	Total(N)	Univariate analysis		Multivariate analysis	
		HR (95% CI)	*P* value	HR (95% CI)	*P* value
**T stage(T3&T4 vs T2)**	**492**	**3.785 (2.140-6.693)**	**< 0.001**	**1.444 (0.690-3.023)**	**0.330**
**N stage(N1 vs N0)**	**426**	**1.946 (1.202-3.150)**	**0.007**	**0.981 (0.568-1.695)**	**0.945**
**M stage(M1 vs M0)**	**458**	**3.566 (0.494-25.753)**	**0.208**		
**Gleason score(9&10 vs 6&7&8)**	**499**	**4.590 (3.038-6.934)**	**< 0.001**	**2.375 (1.382-4.082)**	**0.002**
**PSA(ng/ml)(≥4 vs < 4)**	**442**	**4.196 (2.095-8.405)**	**< 0.001**	**1.814 (0.813-4.050)**	**0.146**
**Age(> 60 vs ≤ 60)**	**499**	**1.302 (0.863-1.963)**	**0.208**		
**Primary therapy outcome(PD&SD&PR vs CR)**	**438**	**6.627 (4.337-10.126)**	**< 0.001**	**3.586 (2.026-6.348)**	**< 0.001**
**Race(Asian&White vs Black&African American)**	**484**	**1.728 (0.866-3.448)**	**0.120**		
**Residual tumor(R1&R2 vs R0)**	**468**	**2.365 (1.566-3.570)**	**< 0.001**	**0.928 (0.546-1.578)**	**0.783**
**Zone of origin(Overlapping & Multiple Zones vs Peripheral &Central &Transition Zone)**	**275**	**1.318 (0.819-2.123)**	**0.256**		
**MAPK8IP2(High vs Low)**	**499**	**2.134 (1.392-3.272)**	**< 0.001**	**1.690 (1.045-2.733)**	**0.032**

*HR* Hazard ratio, *CI* Confidence interval, *PSA* Prostate specific antigen, *PD* Progressive disease, *SD* Stable disease, *PR* Partial response, *CR* Complete response

**Table 3:** Univariate and multivariate Cox regression analyses of the factors predict overall survival in PCa

Factors	Total(N)	Univariate analysis		Multivariate analysis	
		HR (95% CI)	*P* value	HR (95% CI)	*P* value
T stage(T3&T4 vs T2)	**492**	**3.294 (0.612-17.727)**	**0.165**		
**N stage(N1 vs N0)**	**426**	**3.516 (0.778-15.896)**	**0.102**		
**M stage(M1 vs M0)**	**458**	**59.383 (6.520-540.817)**	**< 0.001**	**89.364 (3.202-2494.022)**	**0.008**
**Gleason score(9&10 vs 6&7&8)**	**499**	**4.842 (1.206-19.436)**	**0.026**	**0.596 (0.090-3.961)**	**0.592**
**PSA(ng/ml)(≥4 vs < 4)**	**442**	**10.479 (2.471-44.437)**	**0.001**	**3.116 (0.458-21.202)**	**0.245**
**Age(> 60 vs ≤ 60)**	**499**	**1.577 (0.440-5.648)**	**0.484**		
**Primary therapy outcome(PD&SD&PR vs CR)**	**438**	**8.999 (1.813-44.681)**	**0.007**	**5.987 (0.828-43.301)**	**0.076**
**Race(Asian&White vs Black&African American)**	**484**	**1.451 (0.270-7.794)**	**0.665**		
**Residual tumor(R1&R2 vs R0)**	**468**	**2.598 (0.696-9.694)**	**0.155**		
**Zone of origin(Overlapping & Multiple Zones vs Peripheral &Central &Transition Zone)**	**275**	**2.005 (0.531-7.578)**	**0.305**		
**MAPK8IP2(High vs Low)**	**499**	**13.681 (1.647-113.629)**	**0.015**	**11.964 (1.184-120.873)**	**0.035**

*HR* Hazard ratio, *CI* Confidence interval, *PSA* Prostate specific antigen, *PD* Progressive disease, *SD* Stable disease, *PR* Partial response, *CR* Complete response

Next, we used ROC curves to evaluate the ability of MAPK8IP2 to discriminate between PCa sample and non-PCa samples. Figure 2I shows that MAPK8IP2 can serve as a diagnostic biomarker to distinguish PCa from non-PCa tissues with a certain accuracy (AUC = 0.814, CI: 0.779-0.850).

### MAPK8IP2 genetic alteration in PCa patients

**Fig. 3:**
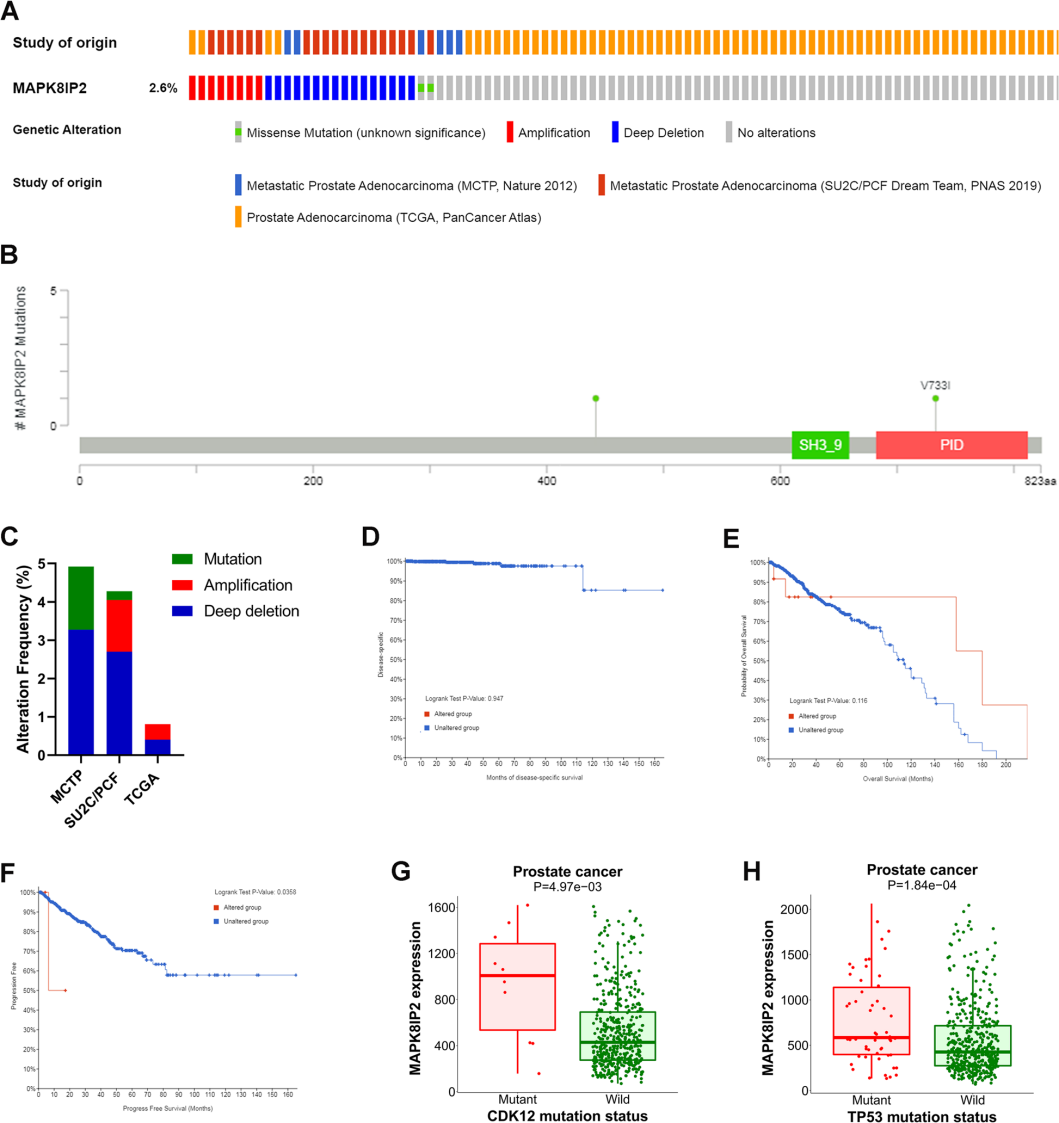
Genetic alteration of MAPK8IP2 in PCa (cBioPortal). **A** OncoPrint visual summary of MAPK8IP2 alteration. **B** Schematic representation of MAPK8IP2 mutations. **C** Summary of MAPK8IP2 alterations in PCa from the MCTP, Nature 2012; SU2C/PCF Dream Team, PNAS 2019; and TCGA, PanCancer Atlas. **D**-**F** Kaplan–Meier plotter was used to compare the DSS (**D**), OS (**E**) and PFS (**F**) of patients in the MAPK8IP2 altered and unaltered groups. **G**, **H** The gene mutations of TP53 (**G**) and CDK12 (**H**) alter MAPK8IP2 expression levels. DSS: disease-specific survival; OS: overall survival; PFS: progression-free survival

A total of 999 PCa patients from three datasets containing survival data were evaluated for MAPK8IP2 genetic alterations. The proportion of MAPK8IP2 genetic alteration in PCa was 2.6% (Fig. 3A), which consisted of missense mutation (Fig. 3B), amplification, and deep deletion were 0.2% (2/999), 0.8% (8/999), and 1.6% (16/999), respectively. In three datasets (MCTP, Nature 2012; SU2C/PCF Dream Team, PNAS 2019; and TCGA, PanCancer Atlas), the MAPK8IP2 genetic alteration rates were 0.81% (4/494), 4.28% (19/444) 4.92% (3/61), respectively (Fig. 3C). Kaplan–Meier plotters showed that MAPK8IP2 altered group was correlated with poor progression-free survival (PFS) (log-rank test: *P* = 0.0358) (Fig. 3F), but was not significantly correlated with disease specific survival (DSS) (Fig. 3D) and OS (Fig. 3E). In addition, through the muTarget platform, MAPK8IP2 mutations did not affect the expression of other genes, but the CDK12 and TP53 mutation groups were found to have higher MAPK8IP2 expression levels than the wild-type group (Fig. 3G, H).

### DNA methylation of MAPK8IP2 in PCa patients

**Fig. 4:**
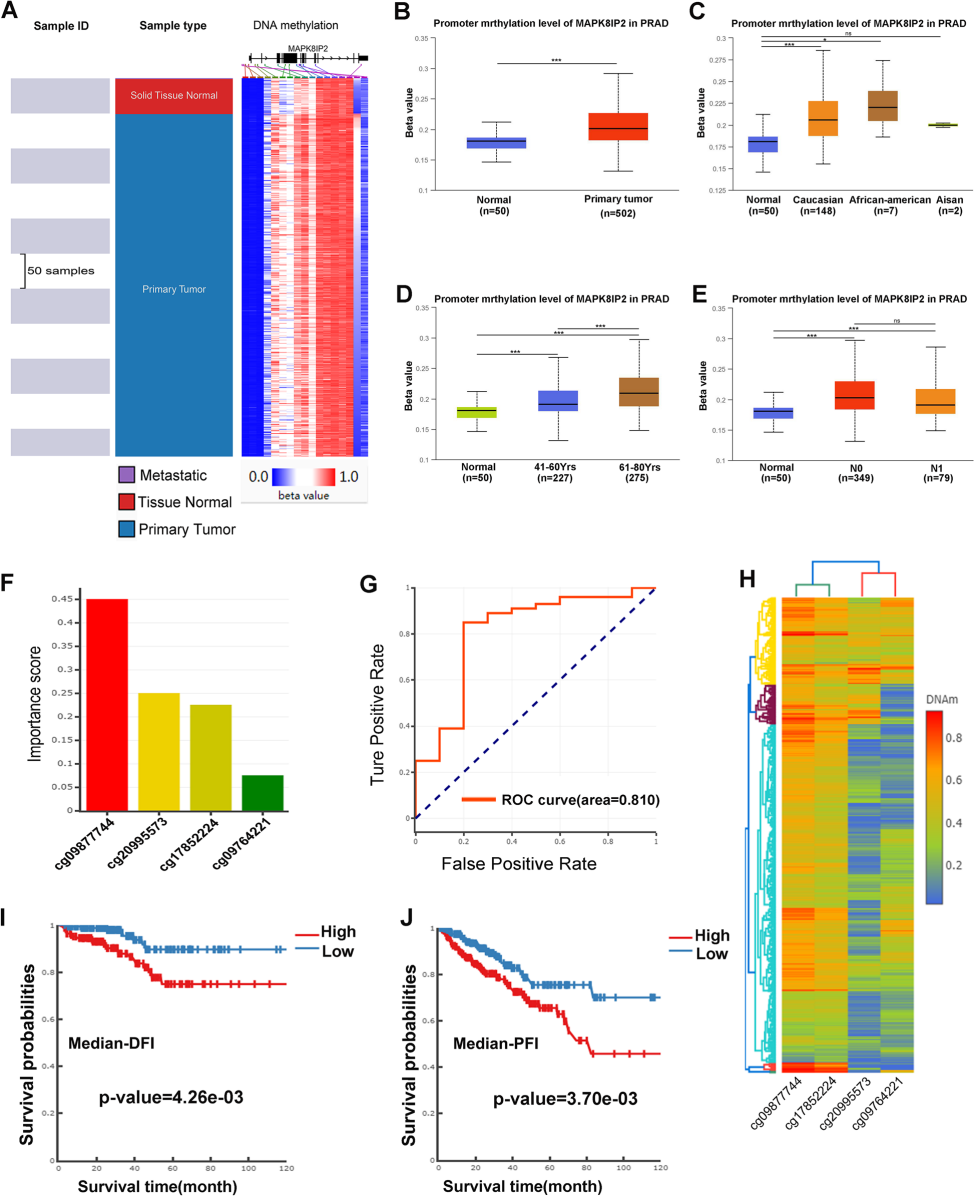
DNA methylation of MAPK8IP2 is strongly associated with PCa. **A** Heatmap comparing the DNA methylation levels of MAPK8IP2 between normal tissue and primary tumor tissue (UCSC Xena). **B**-**E** MAPK8IP2 promoter methylation profile based on sample type (**B**), patient race (**C**), patient age (**D**), and nodal metastasis status (**E**) (UALCAN). **F**-**H** Diagnostic model for PCa based on CpG sites located within MAPK8IP2, the model includes a bar plot of important score (**F**), ROC curve (**G**), and clustering heatmap of importance CpG sites (**H**) (DNMIVD). **I**, **J** Kaplan–Meier curves comparing DFI (**I**),and PFI (**J**) in the low and high groups of DNA methylation of MAPK8IP2 (DNMIVD). ROC: receiver operating characteristic; DFI: disease free interval; PFI: progression-free interval. *p < 0.05, **p < 0.01, ***p < 0.001

We used the UCSC Xena and UALCAN databases to compar the DNA methylation levels of MAPK8IP2 between normal and tumor tissues in PCa. The heat map (Fig. 4A) suggested that the methylation levels of most CpG sites in primary tumors were higher than those in normal tissues, and the box plot (Fig. 4B) analysis also yielded the same result that primary tumor tissues had higher MAPK8IP2 DNA methylation levels than normal tissues. Subgroup analysis indicated that MAPK8IP2 DNA methylation levels were also upregulated in various groups based on race, age, and nodal metastasis status (Fig. 4C-E).

We further used the DNMIVD database to investigate the diagnostic value of CpG sites located within MAPK8IP2 to distinguish tumor samples from normal samples and to perform survival analysis of PCa patients in the low and high groups of DNA methylation of MAPK8IP2. The diagnostic model (Fig. 4F-H) results showed that four CpG sites (cg09877744, cg20995573, cg17852224, and cg09764221) were identified as potential markers for the diagnosis of PCa (the AUC of the ROC curve was 0.810, Fig. 4G), and cg09877744 had the highest importance score (bar plot, Fig. 4F) and DNA methylation levels (clustering heatmap, Fig. 4H). Kaplan–Meier survival analysis showed that the high MAPK8IP2 methylation group had a shorter disease free interval (DFI) (*P* = 4.26e-03, Fig. 4I) and PFI (*P* = 3.70e-03, Fig. 4J) than the low MAPK8IP2 methylation group.

### Relationship between MAPK8IP2 expression and the expression of m6A RNA methylation related genes in PCa

**Fig. 5:**
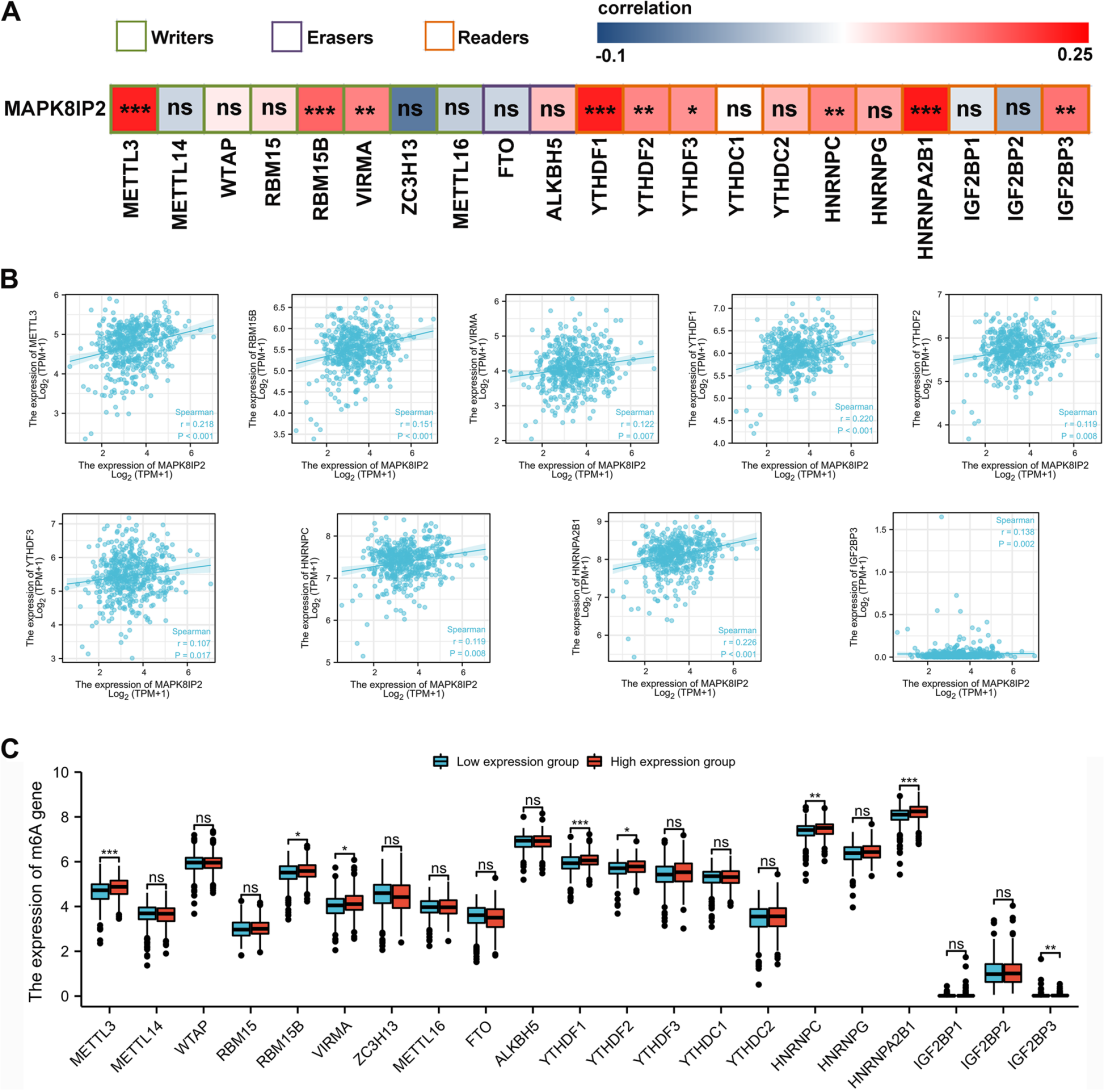
The correlations between MAPK8IP2 expression and the expression of m6A RNA methylation-related genes in PCa (TCGA-PRAD). **A** The correlation of MAPK8IP2 expression levels with m6A RNA methylation gene expression levels in PCa. **B** Scatter plots show a significant correlation between MAPK8IP2 expression and m6A-related genes. These genes include METTL3, RBM15B, VIRMA, YTHDF1, YTHDF2, HNRNPC, HNRNPA2B1, and IGF2BP3. **C** Differential expression of m6A-related genes in the low and high MAPK8IP2 expression groups. *p < 0.05, **p < 0.01, ***p < 0.001

The TCGA PRAD dataset was used to analyze the association between MAPK8IP2 expression and 21 m6A RNA methylation-related genes in PCa. The results showed that 9 m6A-related genes correlated with MAPK8IP2 expression (Fig. 5A). The scatter plot (Fig. 5B) shows 9 significantly positively correlated m6A-related genes including METTL3 (*r* = 0.218, *P* < 0.001), RBM15B (*r* = 0.151, *P* < 0.001), VIRMA (*r* = 0.122, *P* = 0.007), YTHDF1 (*r* = 0.220, *P* < 0.001), YTHDF2 (*r* = 0.119, *P* = 0.008), YTHDF3 (*r* = 0.107, *P* = 0.017), HNRNPC (*r* = 0.119, *P* = 0.008), HNRNPA2B1 (*r* = 0.226, *P* < 0.001), and IGF2BP3 (*r* = 0.138, *P* = 0.002). Then, a total of 499 samples were divided into low (*n* = 249) and high (*n* = 250) MAPK8IP2 expression group. We compared the expression of 21 m6A-related genes between the low and high MAPK8IP2 expression groups, and found significantly increased expression of METTL3, RBM15B, VIRMA, YTHDF1, YTHDF2, HNRNPC, HNRNPA2B1, and IGF2BP3 in the high expression group (*P* < 0.05) (Fig. 5C).

### Correlation between MAPK8IP2 expression and immune infiltration in PCa

**Fig. 6:**
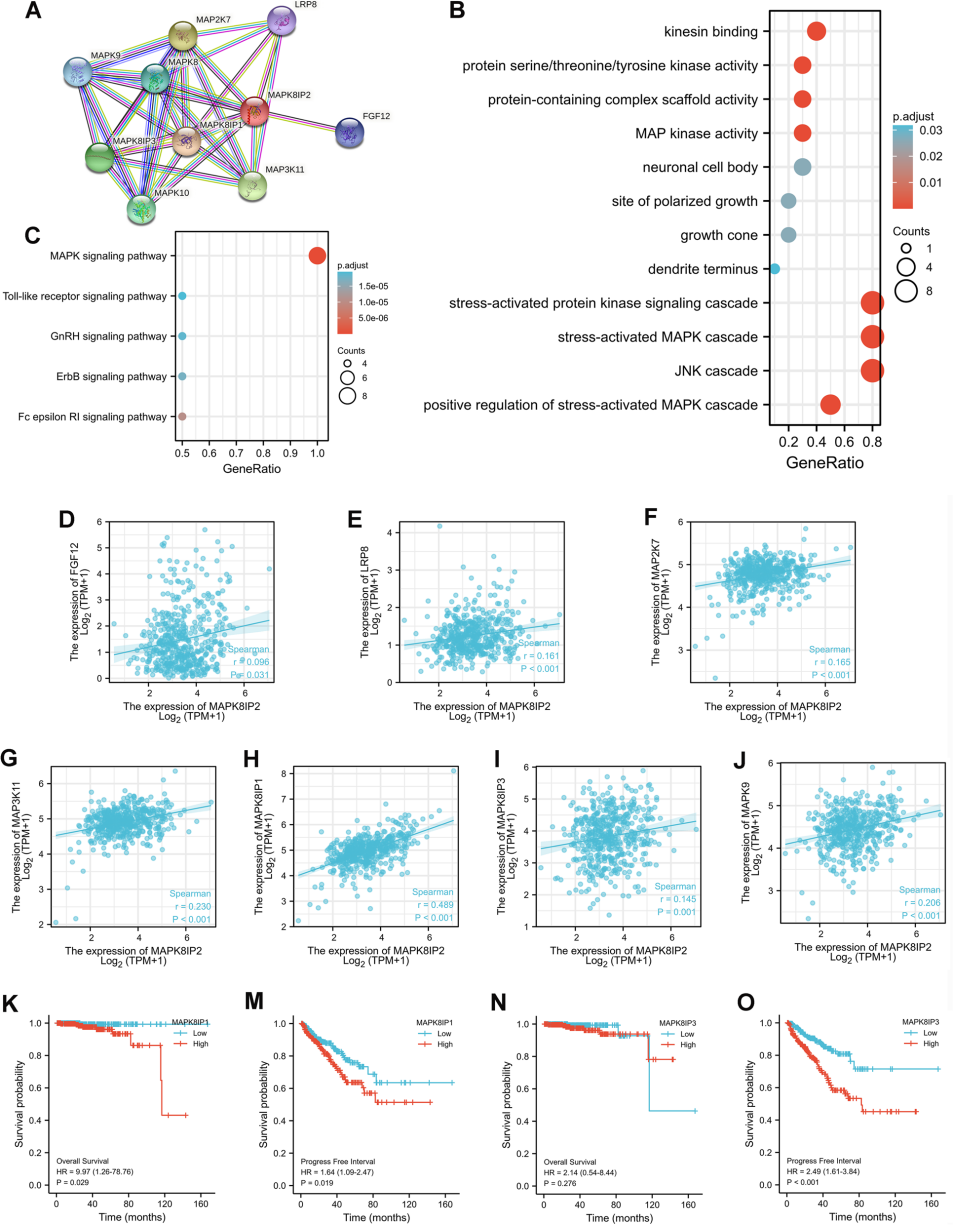
Association of MAPK8IP2 expression with immune cell infiltration in prostate cancer. **A** Forest plotters showed the relationship between MAPK8IP2 expression and immune cells of 24 types. **B**-**E** The difference in B cells (**B**), Mast cells (**C**), Th1 cells (**D**), and T helper cells (**E**) infiltration levels in the low and high MAPK8IP2 expression groups. **F**-**I** Scatter plotters show the correlation between MAPK8IP2 expression levels and B cells (**F**), Mast cells (**G**), Th1 cells (**H**), and T helper cells (**I**) infiltration levels. *p < 0.05, **p < 0.01

We analyzed the correlation of MAPK8IP2 expression with immune infiltration by using the ssGSEA method. As shown in Fig. 6A, there was a negatively correlation between MAPKJ8IP2 expression and the infiltration levels of most immune cells. Then, a total of 499 samples patients from the TCGA PRAD cohort were divided into low (*n* = 249) and high (*n* = 250) MAPK8IP2 expression groups. We determined the correlation coefficients between the levels of different immune cell types and MAPK8IP2 expression with |r| > 0.1 as the cut off and found that the infiltration levels of B cells, mast cells, Th1 cells and T helper cells were significantly lower in the high expression group (*P* < 0.05) (Fig. 6B-E). A scatter plot was used to display the correlations between MAPK8IP2 expression and the infiltration levels of 4 types of immune cells (Fig. 6F-I).

### MAPK8IP2-interacting protein analysis and GO and KEGG enrichment analysis

**Fig. 7:**
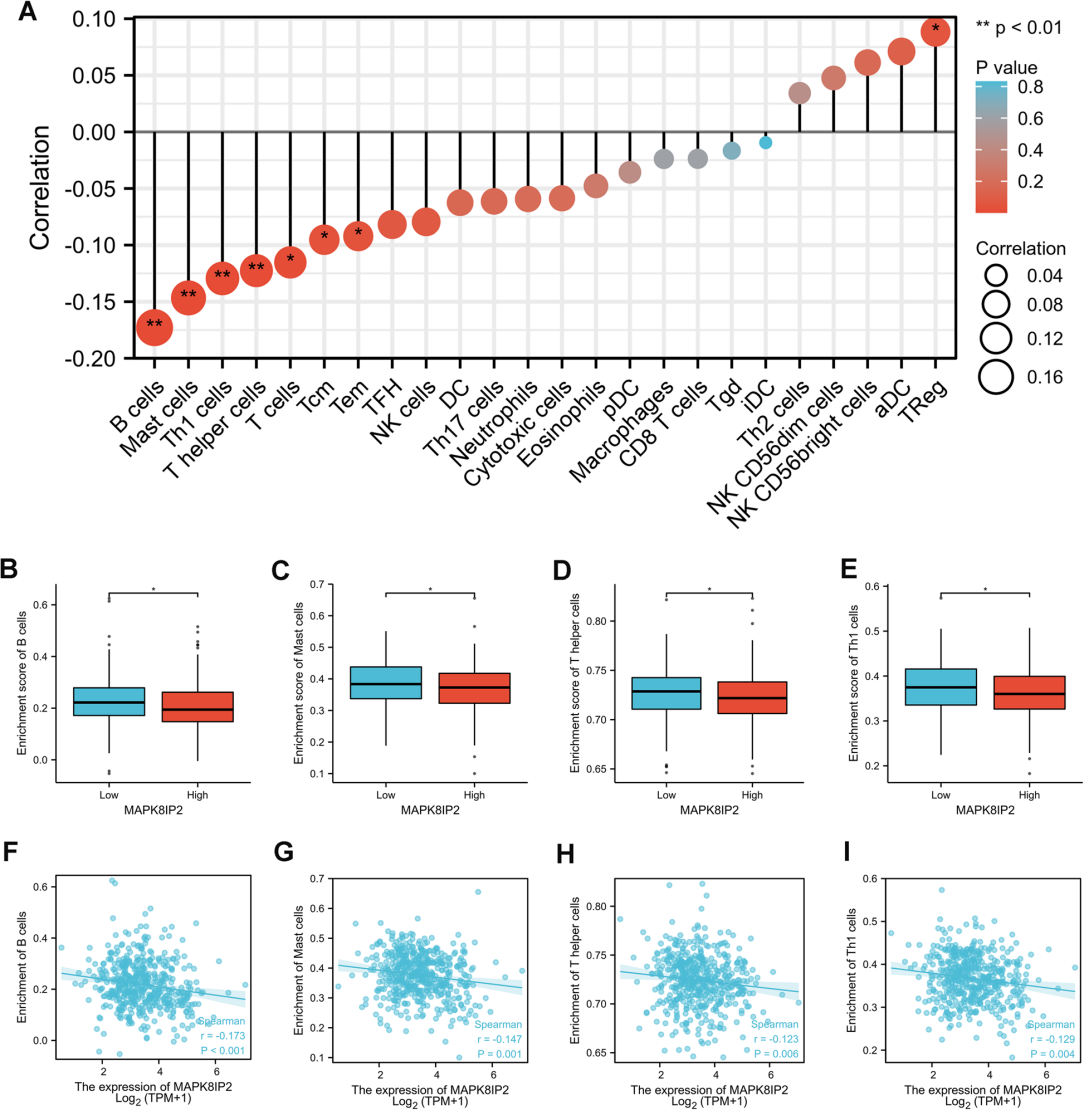
Enrichment analysis of MAPK8IP2 Protein-Protein Interaction in PCa. **A** MAPK8IP2 interaction proteins network in PCa (STRING database). **B** Gene Ontology (GO) term enrichment analysis. **C** Kyoto Encyclopedia of Genes and Genomes (KEGG) enrichment analysis (http://www.kegg.jp/kegg/kegg1.html). **D**-**J** Scatter plots showed a significant positive correlation between MAPK8IP2 and seven partner genes. **K**, **M** Kaplan–Meier curves comparing OS (**K**), and PFI (**M**) in the low and high MAPK8IP1 expression groups. **N**, **O** Kaplan–Meier curves comparing OS (**N**), and PFI (**O**) in the low and high MAPK8IP3 expression groups. OS: overall survival; PFS: progression-free survival

We constructed the PPI network of MAPK8IP2 via the STRING database, and the top 9 functional partner genes were identified: MAPK8IP1, MAP2K7, MAP3K11, MAPK8IP3, MAPK10, MAPK8, MAPK9, FGF12, and LRP8 (Fig. 7A). GO analysis showed that MAPK8IP2 and partner genes are mainly involved in kinesin binding, neuronal cell body, growth cone, and stress-activated protein kinesin signaling cascade (Fig. 7B). KEGG analysis revealed that MAPK8IP2 and partner genes were mainly correlated with the MAPK signaling pathway (Fig. 7C). Then, the correlation between MAPK8IP2 and 9 partner genes was analyzed. The results showed that MAPK8IP2 expression was significantly positively correlated with the expression of FGF12, LRP8, MAP2K7, MAP3K11, MAPK8IP1, MAPK8IP3, and MAPK9 (Fig. 7D-J). We further performed Kaplan–Meier survival analysis for MAPK8IP2-related genes, grouped by median value. The results showed that the MAPK8IP2 related genes not significant difference in survival, besides MAP2K7, MAP3K11, MAPK8IP1 and MAPK8IP3. As shown in Fig. 7K-O and Fig. S1A-D, the high MAPK8IP1 expression group had poor OS (Fig. 7K) and PFI (Fig. 7M), and the high MAP2K7, MAP3K11, and MAPK8IP3 expression groups had a short PFI (Fig. S1A, C; Fig. 7O) but no significant difference in OS (Fig. S1B, D; Fig. 7N). In addition, we further analyzed the expression of MAPK8IP2 and its partner genes through public databases. Univariate and multivariate Cox regression analyses were performed on MAPK8IP2 and its partner genes (MAPK8IP1, MAPK8IP3, MAP2K7, and MAP3K11), and the results showed that MAPK8IP2 and MAPK8IP3 were significantly associated with the progression-free interval of PCa patients (Table S4). Then, the differentially expressed genes correlated with MAPK8IP2 and MAPK8IP3 were analyzed via the LinkedOmics database. We identified MAPK8IP2 and MAPK8IP3 related genes by Spearman correlation analysis (Fig. S4A, D). The heatmaps show the top 50 positively correlated genes and the top 50 negatively correlated genes (Fig. S4B, C, E, F). We selected those with positive Spearman correlation coefficients > 0.4 for the analysis and found that only PDIA2 was positively correlated with both MAPK8IP1 and MAPK8IP3 (Fig. S4G).

### SiRNA-MAPK8IP2 transfection inhibited the proliferation, migration, and invasion of PCa cells in vitro

**Fig. 8:**
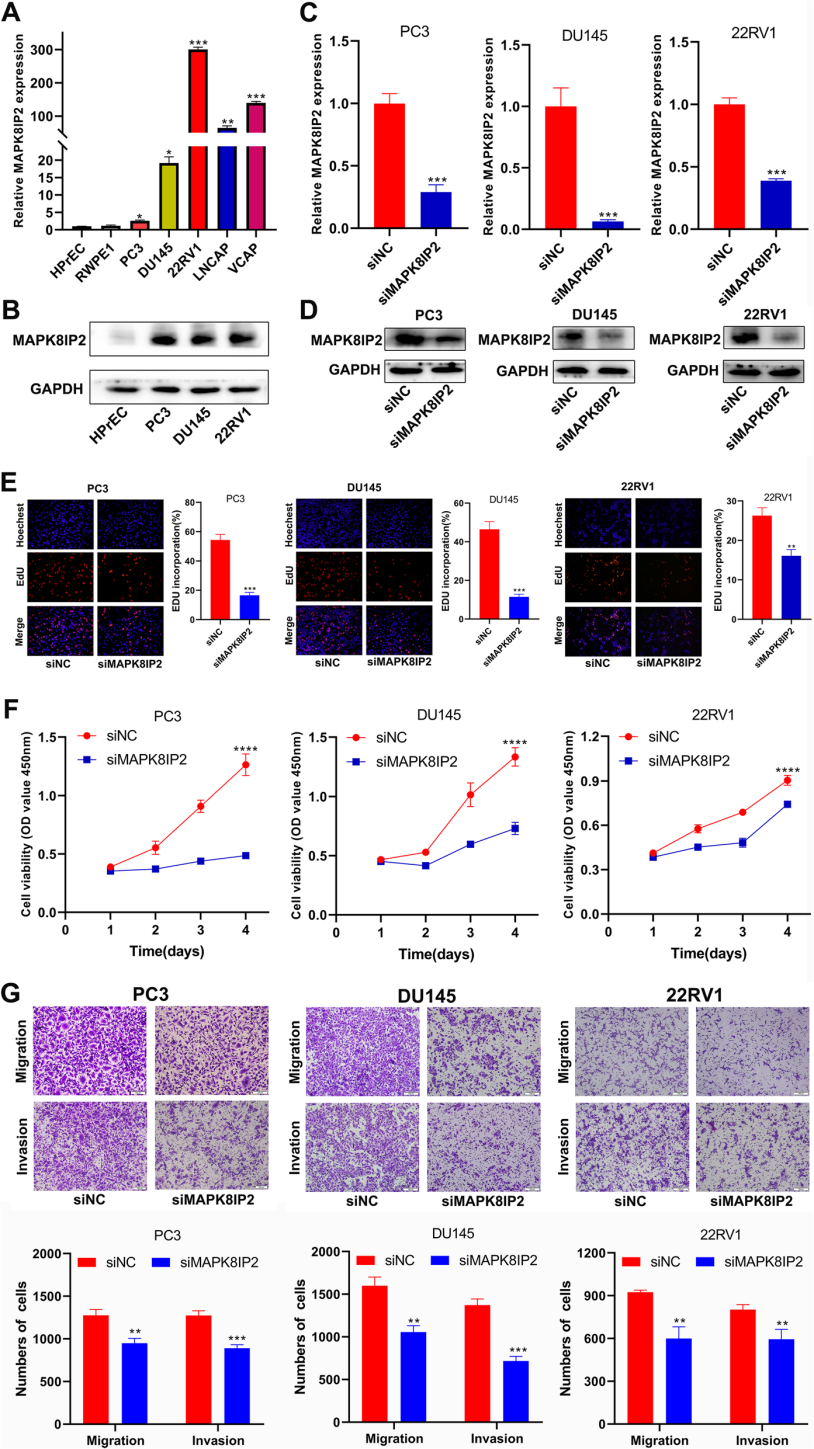
MAPK8IP2 is highly expressed in PCa cells and knockdown of MAPK8IP2 inhibited PC3, DU145, and 22RV1 cells proliferation, migration, and invasion. **A** qRT–PCR analysis showed high expression of MAPK8IP2 in PCa cells compared with normal prostate epithelial cell (HPrEC and RWPE1). **B** Western blot analysis demonstrated that MAPK8IP2 highly expressed in PC3, DU145, and 22RV1 cells compared with HPrEC cells. **C**, **D** qRT–PCR (**C**) and Western blot (**D**) detection of the MAPK8IP2 expression level after transfection with siNC and siMAPK8IP2. **E**, **F** EdU (**E**) and CCK-8 (**F**) assays revealed that knockdown of MAPK8IP2 suppressed PC3, DU145, and 22RV1 cells growth. **G** PC3, DU145, and 22RV1 cells migration and invasion abilities were assessed by transwell assays after downregulation of MAPK8IP2. *p < 0.05, **p < 0.01, ***p < 0.001, ****p < 0.0001

The expression levels of MAPK8IP2 were detected by qRT–PCR, and we found that MAPK8IP2 mRNA expression levels were significantly higher in PCa cell lines (PC3, DU145, 22RV1, LNCAP, and VCAP) than in normal prostate epithelial cells (HPrEC and RWPE1) (Fig. 8A). Western blot analysis demonstrated that MAPK8IP2 protein was highly expressed in PC3, DU145, and 22RV1 cell lines compared to HPrEC cell line (Fig. 8B).

Therefore, we further evaluated the biological function of MAPK8IP2 in PCa cells, and MAPK8IP2 specific siRNA was transfected into PC3, DU145, and 22RV1 cells. MAPK8IP2 mRNA and protein expression levels were significantly downregulated after transfection with siRNA-MAPK8IP2 (Fig. 8C, D). EdU and CCK-8 assays showed that downregulation of MAPK8IP2 efficiently inhibited the growth of PC3, DU145, and 22RV1 cells (Fig. 8E, F). Then, transwell assays were performed to assess the effect of MAPK8IP2 on PC3, DU145, and 22RV1 cells migration and invasion. The results revealed that knockdown of MAPK8IP2 significantly suppressed the migration and invasion capabilities of PC3, DU145, and 22RV1 cells (Fig. 8G).

## Discussions

Studies have demonstrated that JNK and P38 MAPK signaling are closely related to human cancers. MAPK8IP2 is member of the JIP group of MAPK scaffolding proteins and can interact with JNK and p38 [6, 28, 29]. We therefore considered that MAPK8IP2 may play an important role in the development of cancer.

In this study, we found that MAPK8IP2 was upregulated in most cancer tissues by analyzing the TCGA and GTEx cohorts of the UCSC database. Based on the TCGA-PRAD and GSE70768 datasets, we confirmed that MAPK8IP2 expression in PCa tissues was significantly higher than that in normal tissues (paired or unpaired). High expression of MAPK8IP2 was associated with adverse clinicopathological factors and with poor survival in PCa patients. Multivariate Cox analysis demonstrated that MAPK8IP2 expression level was a significant independent prognostic factor terms of OS and PFI. The ROC curve analysis showed that MAPK8IP2 had high diagnostic value for PCa.

Genetic alterations are closely correlated with cancers. Several genetic alterations have been shown to be associated with the initiation and development of PCa [30]. We found that the MAPK8IP2 genetic alteration frequency was only 2.6% in PCa, mainly deep deletion. Although MAPK8IP2 genetic alteration was not significantly correlated with DSS and OS, MAPK8IP2 altered group had significantly poorer PFS than the unaltered group. Considering the small sample size of the MAPK8IP2 altered group, more data are needed for validation. In addition, we found that the CDK12 and TP53 mutation groups had higher expression levels of MAPK8IP2 than the wild-type group. A previous study demonstrated that advanced PCa patients with CDK12 mutations had worse clinical characteristics and were more likely to progress [31]. Patients with metastatic castration-resistant prostate cancer with CDK12 mutations had a worse prognosis than those without CDK12 mutations [32]. TP53 is also one of the most commonly mutated genes in prostate cancer. TP53 mutations were correlated with a poor outcome in PCa patients [33]. The detection of TP53 alterations has clinical value for guiding the precise treatment of hormone-native prostate cancer [34]. These results showed that the prognosis of PCa patients was closely associated with MAPK8IP2 genetic mutation and MAPK8IP2-related genetic mutation.

DNA methylation is a common epigenetic mechanism, and aberrant DNA methylation is strongly associated with cancer. In PCa, aberrant methylation patterns are correlated with tumorigenesis and progression [35, 36]. In this study, we discovered that MAPK8IP2 methylation levels in prostate cancer tissues were higher than in normal tissues. In PCa, survival was worse in the high methylation level group than in the low methylation level group of MAPK8IP2. The ROC analysis showed that four CpG sites located within MAPK8IP2 have better reliability for diagnosing PCa. A recent study found that the expression level of MAPK8IP2 was significantly negatively correlated with DNA methylation in pancreatic cancer (*R* = -0.507) [10]. However, our results suggest that the expression level of MAPK8IP2 may be positively correlated with DNA methylation in PCa. This regulatory mechanism may differ from the classical transcriptional silencing mechanism of DNA methylation, but is associated with DNA methylation-induced transcriptional activation [37].

m6A methylation is the most abundant epigenetic modification in eukaryotic mRNA. It plays an important role in cancer pathogenesis and development [20]. Li et al. [38] demonstrated that YTHDF2 upregulates pAKT by inducing mRNA degradation and promotes prostate cancer progression. Chen et al. [39] found that silencing METTL3 inhibits the invasion and metastasis of prostate cancer cells. Wen et al. [40] showed that m6A modification of lncRNA NEAT1 promotes bone metastasis in PCa. In this study, we found that MAPK8IP2 expression was significantly positively associated with METTL3, RBM15B, VIRMA, YTHDF1, YTHDF2, YTHDF3, HNRNPC, HNRNPA2B1, and IGF2BP3 expression; we also found significantly increased expression of METTL3, RBM15B, VIRMA, YTHDF1, YTHDF2, HNRNPC, HNRNPA2B1, and IGF2BP3 in the high MAPK8IP2 expression group. We speculate that MAPK8IP2 is likely to be modified by m6A to promote PCa progression.

In the analysis of immune infiltration, we discovered that MAPK8IP2 expression was negatively related to the infiltration of some immune cells, mainly including B cells, mast cells, Th1 cells, and T helper cells. However, the correlation was not strong enough. The results indicated that immune infiltration may be associated with MAPK8IP2 to promote PCa progression, but is not a major regulatory factor.

In addition, we further performed GO and KEGG analyses on MAPK8IP2 and 9 partner genes. GO and KEGG analyses revealed that these genes are mainly involved in the stress-activated protein kinesin signaling cascade and MAPK signaling pathway. JNK and P38 activation has been shown to promote prostate cancer cell migration and invasion [41–44]. However, they seem to play a dual role in the proliferation of PCa cells. JNK and P38 activation have been reported to promote PCa cells proliferation [45, 46]. Conversely, Xie et al. [47] found that activation of JNK inhibits the proliferation of PCa cells, and Zhang et al. [48] showed that inhibition of P38 promotes PCa cell proliferation. These results indicated that MAPK8IP2 may mediate the JNK and P38 signaling pathways to promote prostate cancer cell proliferation, migration and invasion. Furthermore, Zhao et al. [11] demonstrated that MAPK8IP2 is regulated by E6 to promote cervical cancer progression via the noncanonical WNT pathway. Therefore, we think that MAPK8IP2 may be involved in other noncanonical pathways to promote PCa progression, besides, via the canonical MAPK signaling pathway. We also found that seven partner genes were significantly positively correlated with MAPK8IP2 expression, but only MAP2K7, MAP3K11, MAPK8IP1 and MAPK8IP3 were significantly associated with survival. Overexpression of MAP2K7, MAP3K11, MAPK8IP1 and MAPK8IP3 was also correlated with worse survival in PCa. Further multivariate Cox regression analysis showed that MAPK8IP2 and MAPK8IP3 were significantly associated with the progression-free interval of PCa patients. MAPK8IP3 like MAPK8IP2 gene belong to the JIPs family and are involved in the regulation of the JNK signaling pathway [49]. Previous studies indicated that MAPK8IP3 may be associated with the malignant progression of prostate cancer [50]. Therefore, we consider that MAPK8IP2and MAPK8IP3 have synergistic effects in promoting PCa progression. The synergistic effect is associated with the PDIA2 gene.

Finally, we confirmed that MAPK8IP2 expression was significantly higher in PCa cell lines than in normal prostate epithelial cells. The proliferation, migration and invasion of prostate cancer cells were inhibited after transfection with siRNA-MAPK8IP2 in vitro.

Several limitations of our research should be recognized. First, potential mechanisms associated with MAPK8IP2 were obtained by bioinformatics analysis, and mechanism validation assays were not performed. Second, we conducted functional assays of PCa cells in vitro, but not in vivo.

## Conclusions

Overall, our study revealed that MAPK8IP2 is highly expressed in PCa. High MAPK8IP2 expression is correlated with a poor prognosis in PCa patients. Furthermore, DNA methylation of MAPK8IP2 and MAPK8IP2 genetic alteration were associated with the prognosis of PCa. MAPK8IP2 might be involved in the regulation of the JNK and P38 MAPK signaling pathways and be modified by m6A. MAPK8IP2 is a promising biomarker for PCa diagnosis, treatment and prognosis evaluation, but more experiments are needed for validation.

## Supplementary Information

**Additional file 1 Figure S1.** Kaplan-Meier curves comparing OS (A, C), and PFI (B, D) in low and high expression, groups of MAP2K7 and MAP3K11. OS: overall survival; PFS: progression-free survival.

**Additional file 2 Figure S2.** Kaplan-Meier curves comparing OS (A, C), and PFI (B, D) in low and high expression groups of FGF12, LRP8 and MAPK9. OS: overall survival; PFS: progression-free survival.

**Additional file 3 Figure S3.** The original images for Western blot in artical. The bands were visualized using the ImageQuant LAS 500.

**Additional file 4 Figure S4.** Differentially expressed genes associated with MAPK8IP2 or MAPK8IP3 in PCa. (A) The correlation between MAPK8IP2 and differentially expressed genes. (B-C) Heat map showing genes positively or negatively correlated with MAPK8IP2 (the top 50 genes). (D) The correlation between MAPK8IP3 and differentially expressed genes. (E-F) Heat map showing genes positively or negatively correlated with MAPK8IP3 (the top 50 genes). (G) The Venn results displayed that only PDIA2 was positively associated with both MAPK8IP2 and MAPK8IP3.

**Additional file 5 Figure S5.** (A) the expression level of MAPK8IP2 was significantly downregulated in the siMAPK8IP2 group compared with the siNC group and the blank group after transfection. (B-D) proliferation migration and invasion of PC3 cells were significantly inhibitor in the siMAPK8IP2 group compared with the siNC group and the blank group.

**Additional file 6 Table S1.** Browse interactions in tabular form**.**

**Additional file 7 Table S2.** Gene Ontology term enrichment analysis of the MAPK8IP2 gene and its partner genes**.**

**Additional file 8 Table S3.** KEGG enrichment analysis of the MAPK8IP2 gene and its partner genes**.**

**Additional file 9 Table S4.** Univariate and multivariate Cox regression analyses of MAPK8IP2 and its partners genes predict progression-free interval in PCa**.**

## Data Availability

The datasets generated or analyzed during this study are available in public database. Public databases are all open access (links are included in the methods section of this article). All data supporting this study are included within the article (and its supplementary information files). All data are available from the corresponding author upon reasonable request.

## Abbreviations

PCa: prostate cancer
MAPK: mitogen-activated protein kinase
JNK: c-Jun NH2-terminal kinase
JIP: JNK-interacting protein
MAPK8IP2: mitogen-activated protein kinase 8 interacting protein 2
GTEx: Genotype-Tissue Expression
HPA: Human Protein Atlas
TCGA: The Cancer Genome Atlas
GEO: Gene Expression Omnibus
ssGSEA: Single Sample Gene Set Enrichment algorithm
PPI: Protein-Protein Interaction
GO: Gene Ontology
KEGG: Kyoto Encyclopedia of Genes and Genomes
qRT-PCR: quantitative real-time PCR
EdU: 5-ethynyl-2′-deoxyuridine
CCK-8: Cell Counting Kit 8
OS: overall survival
PFI: progression-free interval
PFS: progression-free survival
PRAD: prostate adenocarcinoma
