# Supplementary material for: MAPK8IP2 is a potential prognostic biomarker and promote tumor progression in prostate cancer
Source: BMC Cancer. 2022 Nov 11;22:1162. doi: 10.1186/s12885-022-10259-2 (PMC9650804; doi:10.1186/s12885-022-10259-2)
Supplement: Supplementary file 2 — Additional file 2 Figure S2. Kaplan-Meier curves comparing OS (A, C), and PFI (B, D) in low and high expression groups of FGF12, LRP8 and MAPK9. OS: overall survival; PFS: progression-free survival. [file 12885_2022_10259_MOESM2_ESM.pdf]

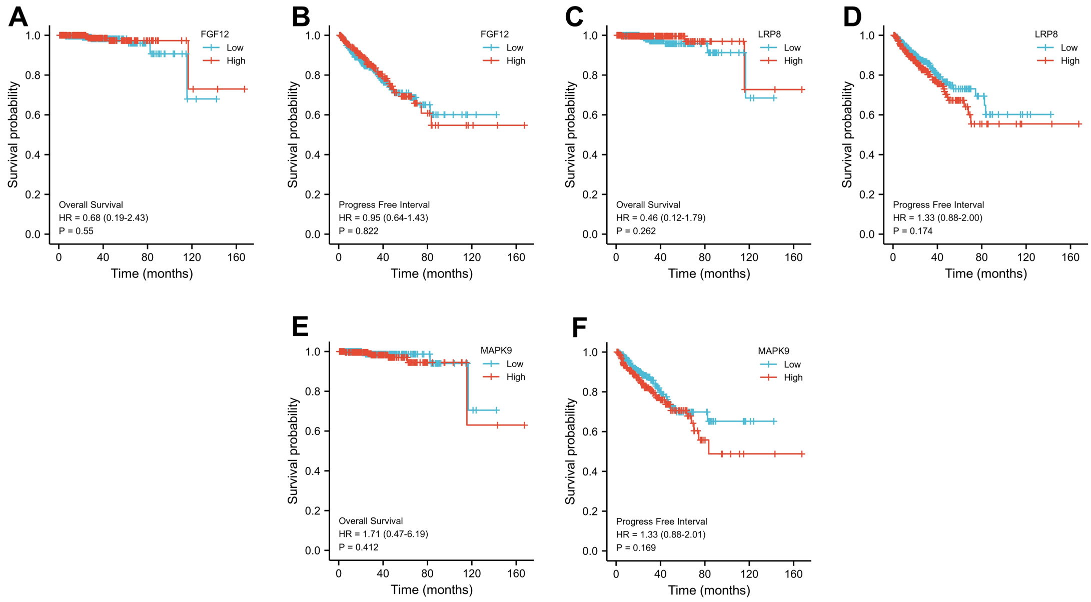

Figure S2 Kaplan-Meier curves comparing OS **(A, C)** , and PFI **(B, D)** in low and high expression groups of FGF12, LRP8 and MAPK9. OS: overall survival; PFS: progression-free survival.
