# Supplementary material for: MAPK8IP2 is a potential prognostic biomarker and promote tumor progression in prostate cancer
Source: BMC Cancer. 2022 Nov 11;22:1162. doi: 10.1186/s12885-022-10259-2 (PMC9650804; doi:10.1186/s12885-022-10259-2)
Supplement: Supplementary file 3 — Additional file 3 Figure S3. The original images for Western blot in artical. The bands were visualized using the ImageQuant LAS 500. [file 12885_2022_10259_MOESM3_ESM.pdf]

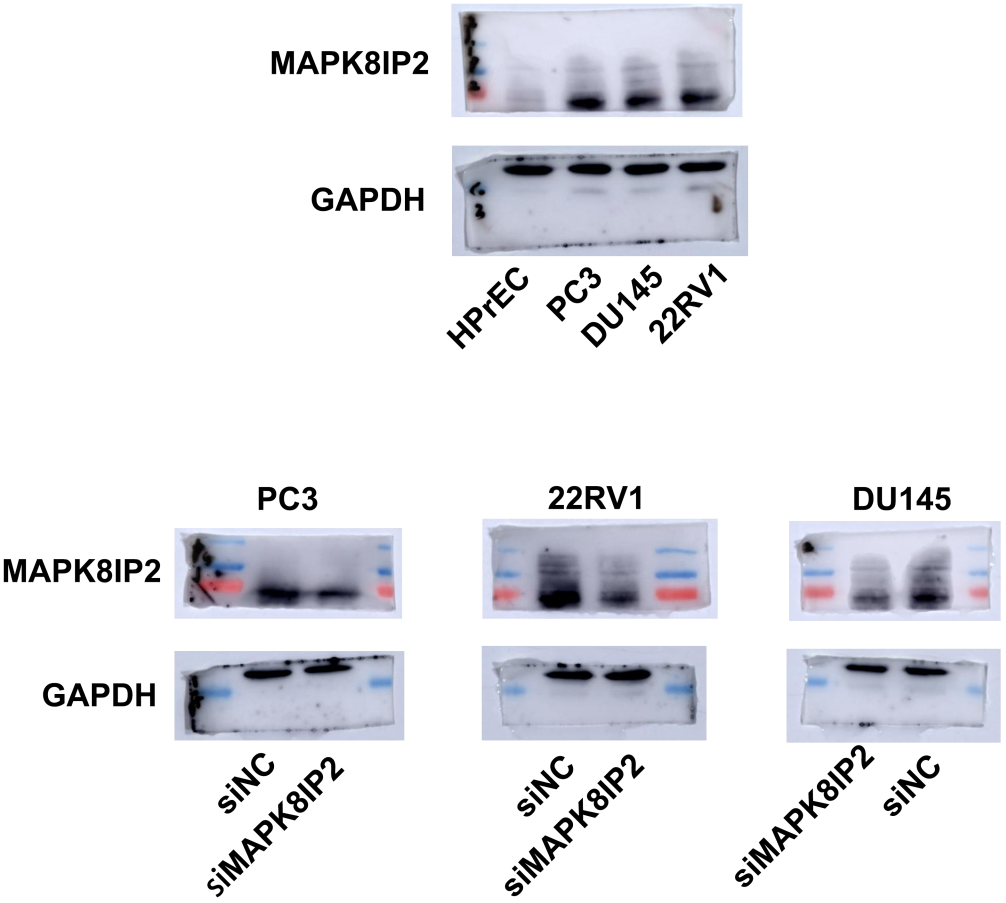

Figure S3 The original images for Western blot in artical. The bands were visualized using the ImageQuant LAS 500 .
