# Supplementary material for: MAPK8IP2 is a potential prognostic biomarker and promote tumor progression in prostate cancer
Source: BMC Cancer. 2022 Nov 11;22:1162. doi: 10.1186/s12885-022-10259-2 (PMC9650804; doi:10.1186/s12885-022-10259-2)
Supplement: Supplementary file 4 — Additional file 4 Figure S4. Differentially expressed genes associated with MAPK8IP2 or MAPK8IP3 in PCa. (A) The correlation between MAPK8IP2 and differentially expressed genes. (B-C) Heat map showing genes positively or negatively correlated with MAPK8IP2 (the top 50 genes). (D) The correlation between MAPK8IP3 and differentially expressed genes. (E-F) Heat map showing genes positively or negatively correlated with MAPK8IP3 (the top 50 genes). (G) The Venn results displayed that only PDIA2 was positively associated with both MAPK8IP2 and MAPK8IP3. [file 12885_2022_10259_MOESM4_ESM.pdf]

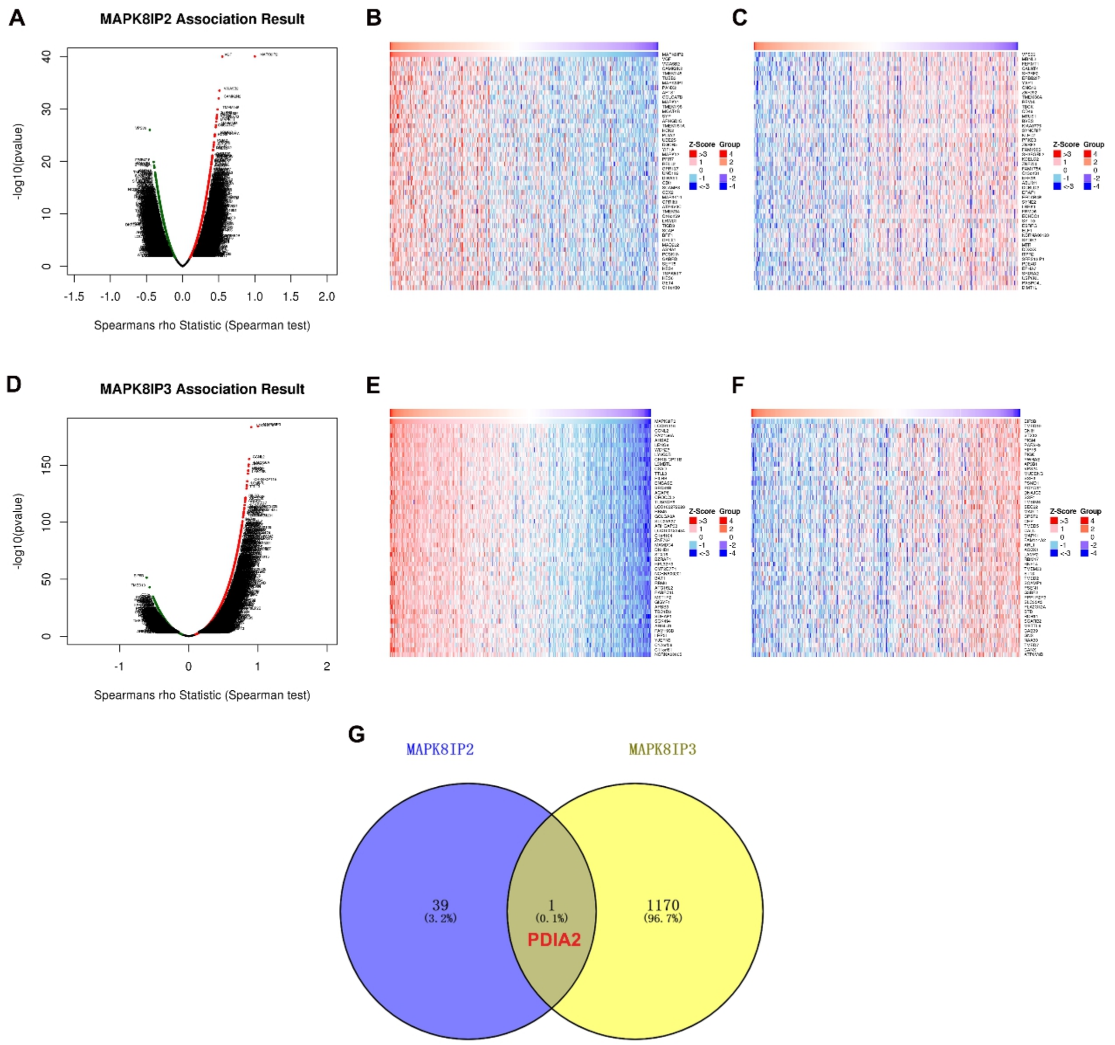

Figure S4 Differentially expressed genes associated with MAPK8IP2 or MAPK8IP3 in PCa. **(A)** The correlation between MAPK8IP2 and differentially expressed genes. **(B-C)** Heat map showing genes positively or negatively correlated with MAPK8IP2 (the top 50 genes). **(D)** The correlation between MAPK8IP3 and differentially expressed genes. **(E-F)** Heat map showing genes positively or negatively correlated with MAPK8IP3 (the top 50 genes). **(G)** The Venn results displayed that only PDIA2 was positively associated with both MAPK8IP2 and MAPK8IP3.
