# Supplementary material for: MAPK8IP2 is a potential prognostic biomarker and promote tumor progression in prostate cancer
Source: BMC Cancer. 2022 Nov 11;22:1162. doi: 10.1186/s12885-022-10259-2 (PMC9650804; doi:10.1186/s12885-022-10259-2)
Supplement: Supplementary file 5 — Additional file 5 Figure S5. (A) the expression level of MAPK8IP2 was significantly downregulated in the siMAPK8IP2 group compared with the siNC group and the blank group after transfection. (B-D) proliferation migration and invasion of PC3 cells were significantly inhibitor in the siMAPK8IP2 group compared with the siNC group and the blank group. [file 12885_2022_10259_MOESM5_ESM.pdf]

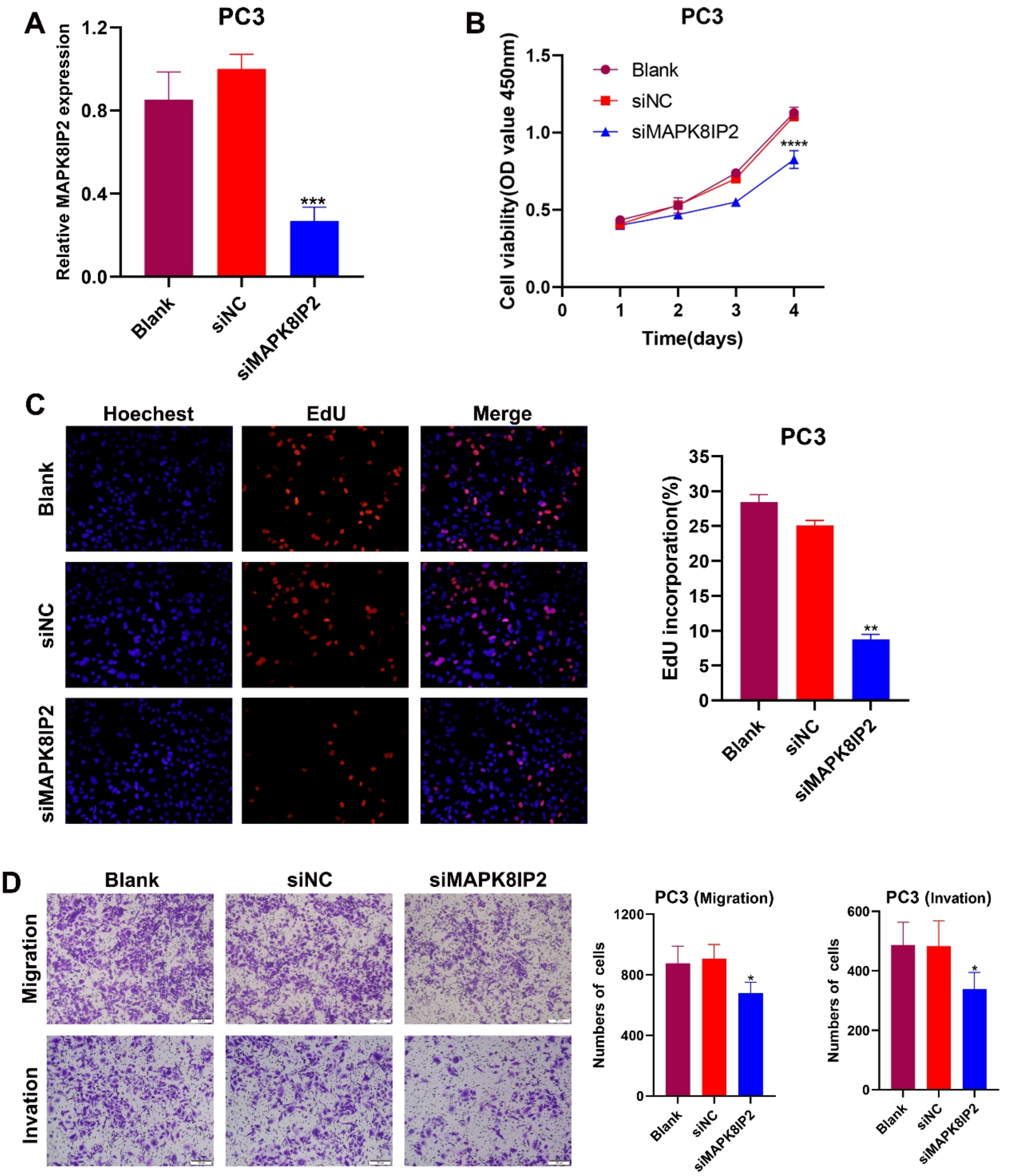

Figure 5S (A) the expression level of MAPK8IP2 was significantly downregulated in the siMAPK8IP2 group compared with the siNC group and the blank group after transfection. **(B-D)** proliferation migration and invasion of PC3 cells were significantly inhibitor in the siMAPK8IP2 group compared with the siNC group and the blank group.
