# Supplementary material for: MAPK8IP2 is a potential prognostic biomarker and promote tumor progression in prostate cancer
Source: BMC Cancer. 2022 Nov 11;22:1162. doi: 10.1186/s12885-022-10259-2 (PMC9650804; doi:10.1186/s12885-022-10259-2)
Supplement: Supplementary file 9 — Additional file 9 Table S4. Univariate and multivariate Cox regression analyses of MAPK8IP2 and its partners genes predict progression-free interval in PCa. [file 12885_2022_10259_MOESM9_ESM.docx]

| Factors | Total(N) | Univariate analysis | |  | Multivariate analysis | |
| --- | --- | --- | --- | --- | --- | --- |
|  |  | Hazard ratio (95% CI) | P value |  | Hazard ratio (95% CI) | P value |
| MAPK8IP1 | 499 | 1.735 (1.184-2.544) | 0.005 |  | 1.086 (0.702-1.680) | 0.712 |
| MAPK8IP2 | 499 | 1.519 (1.233-1.872) | <0.001 |  | 1.365 (1.062-1.755) | 0.015 |
| MAPK8IP3 | 499 | 1.913 (1.472-2.487) | <0.001 |  | 1.743 (1.296-2.344) | <0.001 |
| MAP2K7 | 499 | 2.182 (1.129-4.218) | 0.020 |  | 0.752 (0.362-1.561) | 0.444 |
| MAP3K11 | 499 | 2.937 (1.619-5.328) | <0.001 |  | 1.606 (0.827-3.119) | 0.162 |

**Table S4** Univariate and multivariate Cox regression analyses of MAPK8IP2 and its partners genes predict progression-free interval in PCa

HR:Hazard ratio; CI:confidence interval.
